# SPTAN1 Expression Predicts Treatment and Survival Outcomes in Colorectal Cancer

**DOI:** 10.3390/cancers13143638

**Published:** 2021-07-20

**Authors:** Christopher Schrecker, Sophia Behrens, Rebecca Schönherr, Anne Ackermann, Daniel Pauli, Guido Plotz, Stefan Zeuzem, Angela Brieger

**Affiliations:** 1 Department of Medicine, Biomedical Research Laboratory, University Hospital Frankfurt, Theodor-Stern-Kai 7, 60590 Frankfurt, Germany; sophia.behrens@gmx.net (S.B.); rebecca.schoenherr@stud.pmu.ac.at (R.S.); anne.ackermann@kgu.de (A.A.); daniel.pauli@kgu.de (D.P.); guido.plotz@kgu.de (G.P.); stefan.zeuzem@kgu.de (S.Z.); 2 Faculty of Medicine, Paracelsus Medical University, Strubergasse 21, 5020 Salzburg, Austria

**Keywords:** colorectal cancer, SPTAN1, spectrin, cytoskeleton, metastasis, chemotherapy

## Abstract

**Simple Summary:**

Colorectal cancer (CRC) is a common and deadly form of cancer. Non-erythroid spectrin αII (SPTAN1), a protein of the cytoskeleton, is thought to be involved in CRC development and progression. In this study, we explore whether measuring SPTAN1 levels in resected CRC specimens might help to predict patient survival outcomes and response to chemotherapy. Indeed, we find that higher SPTAN1 protein and mRNA levels in CRC specimens associate with longer patient survival times. Using cell culture experiments, we then show that cells with lower SPTAN1 levels are less susceptible to FOLFOX chemotherapy, a standard treatment regimen for patients with CRC. Overall, our study underscores the importance of cytoskeletal proteins in shaping tumour biology and treatment responses and nominates SPTAN1 as a biomarker to improve patient stratification and refine therapeutic decisions in CRC.

**Abstract:**

Colorectal cancer (CRC) is a leading cause of cancer-related morbidity and mortality. In a cohort of 189 patients with CRC, we recently showed that expression of the cytoskeletal scaffolding protein non-erythroid spectrin αII (SPTAN1) was lower in advanced metastatic tumours. The aim of the present study was to clarify the association of intratumoural SPTAN1 expression levels with treatment and survival outcomes in patients with CRC. The analysis was based on histologic assessment of SPTAN1 protein levels in our own CRC cohort, and transcriptome data of 573 CRC cases from The Cancer Genome Atlas (TCGA). We first establish that high intratumoural levels of SPTAN1 protein and mRNA associate with favourable survival outcomes in patients with CRC. Next, a response prediction signature applied to the TCGA data reveals a possible link between high SPTAN1 transcript levels and improved patient responses to FOLFOX chemotherapy. Complementary in vitro experiments confirm that SPTAN1 knockdown strains of the colon cancer cell lines HT-29, HCT116 mlh1-2 and Caco-2 are less responsive to FOLFOX chemotherapy compared with SPTAN1-proficient control strains. Taken together, we identify SPTAN1 as a novel prognostic biomarker in CRC and show that SPTAN1 expression levels may predict patient responses to chemotherapy. These investigations illustrate how an affordable, histology-based diagnostic test could directly impact therapeutic decision-making at the bedside.

## 1. Introduction

Colorectal cancer (CRC) is a leading cause of cancer-related morbidity and mortality in both men and women [1]. The majority of colorectal tumours are sporadic in nature and arise from a well-defined sequence of somatic mutations termed the adenoma-carcinoma sequence [2]. Conversely, some fifteen per cent of colorectal tumours result from defective DNA mismatch repair (MMR), which ultimately leads to the hypermutable phenotype known as microsatellite instability (MSI) [3]. MSI-high colorectal tumours develop either through germline mutations in one of the MMR genes or by epigenetic silencing of the *MutL homolog 1* (*MLH1*) gene [3]. MSI-high CRCs have a better prognosis and are less likely to metastasise than microsatellite stable CRCs [4,5]. The molecular explanation for their reduced metastatic potential remains unknown.

Our group previously showed that the MMR protein MLH1 is capable of interacting with a number of cytoskeletal scaffolding proteins, including the actin-binding protein non-erythroid spectrin αII (SPTAN1) [6]. Specifically, we found that MLH1-deficient cell lines exhibit reduced levels of SPTAN1 and have impaired migratory ability [7], initially suggesting that loss of SPTAN1 may be partly responsible for the reduced metastatic efficiency of MMR-deficient tumour cells. On the other hand, knockdown of SPTAN1 in CRC cell lines led to reduced cell-cell contact formation [8], indicating that tumour cells expressing low levels of SPTAN1 may detach from their primary tumour and metastasise more easily. Indeed, our recent analysis of 189 patients with CRC showed that SPTAN1 expression is lower in metastatic compared with non-metastatic tumours [8]. These most recent findings strongly implicate loss of SPTAN1 as a driver of invasiveness and metastatic potential.

SPTAN1 is a member of the spectrin family of actin-binding proteins. Spectrins are ubiquitously expressed cytoskeletal scaffolding proteins that crosslink filamentous actin and are involved in various cellular processes including cell adhesion and DNA repair [9]. There are seven different spectrin genes in humans, encoding spectrins alpha I and II, and spectrins beta I to V [9]. In the cytoplasm, spectrins exist as tetramers of two alpha and two beta subunits [9]. Tetramers can bind both actin microfilaments and integral membrane proteins, and thus facilitate formation of an actin meshwork that can be anchored to the plasma membrane [9]. Mutations in the erythroid spectrins αI and βI result in spherocytosis and other red cell disorders [10], whereas aberrant non-erythroid spectrins give rise to defects in neuronal signalling and myocardial excitability, and are known to be involved in neoplastic processes including CRC [9,11].

The aim of this study was to explore the value of SPTAN1 as a predictor of survival and treatment outcomes in CRC. We first compile survival data from our previously characterised CRC cohort and show that patient survival is linked to SPTAN1 protein levels in colorectal tumours. We then analyse gene expression data from The Cancer Genome Atlas (TCGA) to corroborate our findings at the transcriptional level, and to further delineate the association of SPTAN1 mRNA levels with epithelial cohesion, colorectal tumour invasiveness, and patient responses to FOLFOX chemotherapy. Finally, we generate SPTAN1 knockdown strains of three widely used colon cancer cell lines, to further probe the impact of differential SPTAN1 expression levels on treatment responses to FOLFOX chemotherapy.

## 2. Materials and Methods

### 2.1. Patients and Ethical Approval

We included 182 patients with available survival data from our previously published cohort of 189 patients with CRC [8]. All patients underwent colonic resection with curative intent, and without prior exposure to neoadjuvant therapies. Resections were carried out between January 2011 and December 2016 at the University Hospital Frankfurt, a German tertiary academic centre. Data closure for the survival data was 30 April 2019. The study was approved by the local ethics committee, and all patients provided written informed consent. Details of the clinical and survival data compiled for this study have been summarised in Table S1.

### 2.2. Protein Analysis

As part of our previously published study, SPTAN1 protein levels in the 182 colorectal tumours were quantified by digital image analysis of immunohistochemistry stained slides [8]. For the present study, we used the available intensity data and ranked the 182 samples from lowest to highest based on their SPTAN1 staining intensities. The top third (61 patients) with strong staining intensities were defined as the SPTAN1-high group, and the lower two thirds (121 patients) with weak or moderate staining intensities were defined as the SPTAN1-low group. Information on individual tumour staining intensities is shown in Table S1.

### 2.3. Gene Expression Data

Publicly available gene expression data of 573 TCGA colorectal tumours was downloaded from Sage Bionetworks Synapse (syn4978511) in April 2020, along with matching clinical and survival data. TCGA expression data was originally processed and uploaded to Sage Bionetworks in 2015, as part of the collaborative effort by Guinney and colleagues to define and characterise the consensus molecular subtypes of CRC [12]. Details of how the RNA-seq data was processed are provided in the original publication [12]. For the present study, samples were again ranked from lowest to highest, this time according to their SPTAN1 mRNA levels. The top half (286 patients) with high SPTAN1 mRNA levels were again defined as the SPTAN1-high group, and the lower half (287 patients) with low SPTAN1 mRNA levels were defined as the SPTAN1-low group. SPTAN1 mRNA levels for individual tumours are listed in Table S2.

### 2.4. Single-Sample Gene Set Enrichment Analysis

Single-sample gene set enrichment scores were calculated from the pre-processed RNA-seq data (comprising 20,293 genes in 573 samples), for groups of genes involved in actin cytoskeletal function (GO actin cytoskeleton; 503-gene set) and focal adhesion (KEGG focal adhesion; 199-gene set) [13,14]. Enrichment scores were generated for each of the 573 tumour samples, using the default settings of the GenePattern algorithm for single-sample gene set enrichment analysis (ssGSEA) [15]. The scores represent the degree to which the individual genes (in the gene sets of interest) are coordinately up- or downregulated within a given tumour sample. The same GenePattern module was then used to compute immune and stromal enrichment scores, in order to infer the presence of non-tumour cells in the tumour microenvironment of each sample [16]. Details of the algorithm used for ssGSEA can be found at www.genepattern.org (accessed on 30 April 2020) and in the original publication by Barbie and colleagues [15,17].

### 2.5. Gene Set Enrichment Analysis

The 573 TCGA samples were subsequently analysed by gene set enrichment analysis (GSEA), applying SPTAN1 mRNA levels (expressed as z-scores) as a continuous phenotype label. The GenePattern GSEA module [18] was used to interrogate the well-curated Hallmark (50 gene sets) [19], KEGG (186 gene sets) [14] and GO (10,271 gene sets) [13] collections from the Molecular Signatures Database (MSigDB) [19]. We set the collapse dataset parameter to no collapse, and selected the Pearson metric for ranking genes (the default signal-to-noise metric cannot be applied to continuous phenotypes). The analysis was otherwise carried out using the default settings of the GenePattern GSEA module [18]. For each gene set in the analysis, the GSEA algorithm computes a normalised enrichment score (NES), nominal *p*-value, and false discovery rate (FDR) to correct for multiple testing. Again, details of the algorithm used for GSEA can be found at www.genepattern.org (accessed on 30 April 2020) or in the original publication by Subramanian and colleagues [17,18].

### 2.6. Treatment Response Prediction

We applied a qualitative transcriptional signature to the TCGA expression data, in order to predict response to FOLFOX chemotherapy (folinic acid, fluorouracil, and oxaliplatin) for each of the 573 tumour samples. This method compares the relative gene expression levels of five gene pairs (*CALML5*/*IGFBP1*, *CCND2*/*GPR34*, *IRF6*/*WDR75*, *HOXB4*/*SMURF2*, *TRIM11*/*NT5DC3*), and predicts a favourable response to treatment with FOLFOX when the expression level of gene 1 is greater than that of gene 2 in three or more of the five gene pairs [20]. In the original publication, the signature’s area under the ROC curve was 0.94 in the training dataset (correct classification of 62/70 responders and 24/26 non-responders), and 0.82 in the validation dataset (correct classification of 17/20 responders and 4/5 non-responders) [20].

### 2.7. Cell Lines

HT-29 cells (HTB-38), Caco-2 cells (HTB-37), and HEK293T cells (CRL-3216) were purchased from the American Type Culture Collection (Manassas, VA, USA). HCT116 mlh1-2 cells, stably transfected with the pcDNA3.1+/MLH1 overexpression plasmid, were a gift from Professor Franҫoise Praz (Centre National de la Recherche Scientifique in Paris, France). Cells were grown in DMEM (Thermo Fisher Scientific; Waltham, MA, USA) with 10% FBS (Sigma-Aldrich; St. Louis, MO, USA) and 1% penicillin-streptomycin (Sigma-Aldrich). The growth media of stably transduced cell lines was supplemented with puromycin; 5 μg/mL for HT-29 and Caco-2 cells, and 4 μg/mL for HCT116 mlh1-2 cells. All cell lines were regularly tested for mycoplasma, and STR profiling was carried out as recently described [21]. The CRC cell lines used in this study carry different combinations of recurrent genetic and epigenetic alterations: HT-29 cells are *TP53*-mutant, *KRAS* wild-type, *BRAF*-mutant, MSS, CIMP-positive; Caco-2 cells are *TP53*-mutant, *KRAS* wild-type, *BRAF* wild-type, MSS, CIMP-negative; HCT116 mlh1-2 cells are *TP53* wild-type, *KRAS*-mutant, *BRAF* wild-type, MSI, CIMP-positive [22].

### 2.8. Generation of Lentiviral Particles and shRNA Knockdown

The HT-29, HCT116 mlh1-2 and Caco-2 cell lines endogenously express SPTAN1. Stable knockdown of SPTAN1 expression in these cell lines was achieved by transduction with lentivirus encoding interfering MISSION^®^ shRNA nucleic acid molecules, according to the manufacturer’s instructions (Sigma-Aldrich). HEK293T cells were used to generate lentiviral particles. Briefly, 1 × 10^6^ HEK293T cells were plated in 10 cm dishes and co-transfected with 1 μg each of three packaging plasmids (MISSION^®^ pLP1, pLP2, and pLP/VSVG), as well as 3 μg of a lentiviral plasmid vector (MISSION^®^ pLKO.1-puro) encoding one of four short hairpins targeting SPTAN1 mRNA (MISSION^®^ shRNA TRCN0000053669; MISSION^®^ shRNA TRCN00000299160; MISSION^®^ shRNA TRCN00000299159; MISSION^®^ shRNA TRCN00000299161). For the generation of control lentiviral particles, HEK293T cells were co-transfected with 1 μg each of the three packaging plasmids, and 3 μg of a lentiviral plasmid vector encoding non-mammalian shRNA (MISSION^®^ pLKO.1-puro non-mammalian shRNA control plasmid SHC002V). Supernatant containing the lentiviral particles was harvested after 72 h, sterile filtered and used for transduction of CRC cell lines. Target cells were plated at a density of 5 × 10^5^ cells per well in 6-well plates, and fresh lentiviral particles were added three times on day 1, and twice on day 2. From day 3, cells were grown in DMEM culture medium supplemented with puromycin.

### 2.9. Western Blotting

SPTAN1 expression in stably transduced cell lines was quantified by Western blot analysis. Cell lysates were separated on 10% polyacrylamide gels, followed by transfer onto nitrocellulose membranes and antibody detection. Membranes were incubated with primary antibodies targeting SPTAN1 (MAB1622 from Sigma-Aldrich) or β-actin (A5441 from Sigma-Aldrich), followed by a fluorescent labelled secondary antibody (IRDye^®^ 680 LT from LI-COR Biosciences; Lincoln, NE, USA), and then imaged on a FLA-9000 scanner (Fujifilm; Tokyo, Japan). Western blot band intensities were quantified using the image analysis software Multi Gauge version 3.2 (Fujifilm; Tokyo, Japan). Western blots were performed at least three times, as independent biological replicates.

### 2.10. Cell Viability Assay

Response to FOLFOX chemotherapy was assessed by tetrazolium dye (MTT) colorimetric assay. Cells with stable SPTAN1 knockdown were seeded at 1 × 10^4^ cells per well in 96-well culture plates and grown at varying concentrations of FOLFOX chemotherapy (a combination regimen of folinic acid, fluorouracil, and oxaliplatin). The highest treatment dose, FOLFOX 1000×, was prepared to final concentrations of 0.16 μM folinic acid, 0.8 μM fluorouracil, and 0.008 μM oxaliplatin. FOLFOX 100×, 10× and 1× were obtained through serial 10-fold dilutions of FOLFOX 1000×. Cell viability was assessed 12, 24, 48, and 72 h after seeding and treatment initiation. At the specified timepoints, culture media were replaced with media containing the tetrazolium dye MTT (Sigma-Aldrich), at a final concentration of 833 μg/mL, which is enzymatically reduced within cells to insoluble formazan crystals. Following incubation with the dye for 2 h at 37 °C, media were replaced with 100 μL per well of a decolourising solution (DMSO with 0.6% acetic acid and 0.1 g/mL SDS), to extract the insoluble formazan crystals from within cells. After 20 min extraction, the absorbance of the formazan solution was measured at 570 nm using an EnVision^®^ plate reader (PerkinElmer; Waltham, MA, USA). Three independent experiments were carried out, totalling up to 48 individual readings for each combination of cell line, knockdown strain, timepoint and chemotherapy dose.

### 2.11. Statistical Analysis

Data are expressed as mean ± SEM or median and range, as appropriate. Statistical analysis was carried out using the R environment for statistical computing [23]. The survival and survminer packages were used to model patient survival data [24,25]. The relation of SPTAN1 levels and patient outcomes was assessed by simple Cox proportional hazards model, and multivariable models were used to adjust for potential confounders. Population means were compared by analysis of variance (aov function) or unpaired *t*-test (*t*-test function) as appropriate, and Pearson correlation coefficients were computed using the cor.test function. Cell viability of SPTAN1 knockdown and control strains was compared by one-way analysis of variance (aov function), followed by Dunnett’s post-hoc test (using the glht function from the multcomp package) [26]. Results with a *p*-value < 0.05 were considered statistically significant. GenePattern modules include their own statistical analysis as detailed above.

## 3. Results

### 3.1. High Intratumoural SPTAN1 Protein Levels Predict Better Overall Survival in Patients with CRC

First, we considered whether intratumoural SPTAN1 protein levels might be related to survival outcomes in patients with CRC. To address this question, we identified 182 patients with available survival data from our recently published CRC cohort [8]. SPTAN1 protein levels in each of the 182 tumours were analysed by immunohistochemistry as part of our previous study. Staining intensities were determined by digital image analysis and used as a surrogate for SPTAN1 protein levels [8]. For the present study, we ranked the samples from lowest to highest according to their SPTAN1 staining intensities. The top third (61 patients) with strong staining intensities were defined as the SPTAN1-high group, and the lower two thirds (121 patients) with weak or moderate staining intensities were defined as the SPTAN1-low group.

**Figure 1:**
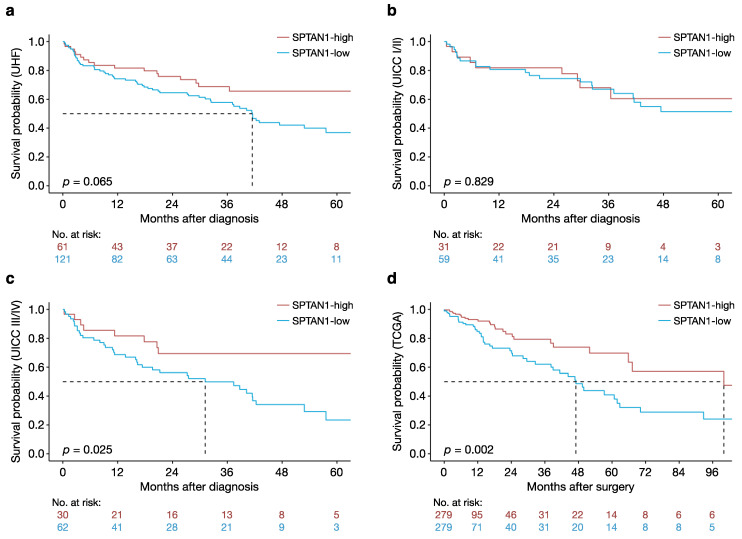
High intratumoural SPTAN1 levels predict better overall survival in patients with CRC. Kaplan-Meier plots depicting the association between overall survival and SPTAN1 protein levels (**a**) in the full cohort from the University Hospital Frankfurt (UHF), and in the subgroups with (**b**) early (UICC stage I/II) or (**c**) advanced (UICC stage III/IV) CRC. The association between overall survival and *SPTAN1* gene expression in the TCGA cohort is shown in (**d**). The number at risk is displayed below each survival curve. Patient survival was assessed from the time of diagnosis in the UHF cohort, and from the time of surgery in the TCGA cohort. Survival data were modelled by proportional hazards regression using the survival and survminer packages in the R environment for statistical computing. Results with a *p*-value < 0.05 were considered statistically significant.

Survival outcomes were analysed by Cox proportional hazards model, in order to explore the potential of intratumoural SPTAN1 protein levels as a survival predictor. Median overall survival was 42 months in the SPTAN1-low group, and was not reached in the SPTAN1-high group (hazard ratio in the SPTAN1-high group, 0.61; 95% confidence interval, 0.36 to 1.03; *p* = 0.065) (Figure 1a). The trend of improved survival in the SPTAN1-high group was maintained after adjusting for patient age and tumour stage in a multivariable proportional hazards model (hazard ratio in the SPTAN1-high group, 0.59; 95% confidence interval, 0.35 to 1.01; *p* = 0.054) (Table 1). It therefore appears that high intratumoural SPTAN1 protein levels may independently predict better overall survival in patients with CRC.

In a subgroup analysis of UICC stage I and II tumours there was no difference in overall survival between the SPTAN1-high and SPTAN1-low groups (hazard ratio in the SPTAN1-high group, 0.92; 95% confidence interval, 0.42 to 2.01; *p* = 0.829) (Figure 1b). Conversely, subgroup analysis of UICC stage III and IV tumours showed improved overall survival in the SPTAN1-high group compared with the SPTAN1-low group (hazard ratio in the SPTAN1-high group, 0.43; 95% confidence interval, 0.21 to 0.90; *p* = 0.025) (Figure 1c). These observations suggest that differential SPTAN1 protein levels are particularly relevant in more advanced colorectal tumours.

### 3.2. High Intratumoural SPTAN1 mRNA Levels Predict Better Overall Survival in Patients with CRC

We next aimed to corroborate our findings at the transcriptional level. To this end, we downloaded publicly available gene expression data of 573 colorectal tumours from The Cancer Genome Atlas (TCGA), along with matching clinical and survival data [12]. We then investigated the relationship between *SPTAN1* gene expression and patient outcomes. Samples were again ranked from lowest to highest, this time according to their SPTAN1 mRNA levels. The top half (286 patients) with high SPTAN1 mRNA levels were again defined as the SPTAN1-high group, and the lower half (287 patients) with low SPTAN1 mRNA levels were defined as the SPTAN1-low group. Survival data were available for 279 patients in each group.

Median overall survival was 100 months in the SPTAN1-high group, and 47 months in the SPTAN1-low group (hazard ratio in the SPTAN1-high group, 0.46; 95% confidence interval, 0.29 to 0.75; *p* = 0.002) (Figure 1d). The clear survival advantage in the SPTAN1-high group was maintained after adjusting for patient age and tumour stage in a proportional hazards model (hazard ratio in the SPTAN1-high group, 0.49; 95% confidence interval, 0.30 to 0.81; *p* = 0.005). Notably, SPTAN1 mRNA levels (expressed as z-scores) remained independently associated with overall survival when treated as a continuous variable (hazard ratio per unit increase in SPTAN1 z-score, 0.77; 95% confidence interval, 0.62 to 0.96; *p* = 0.018; range of SPTAN1 z-score, −5.0 to 2.9). Like high intratumoural SPTAN1 protein levels, high intratumoural SPTAN1 mRNA levels therefore independently predict better overall survival in patients with CRC.

### 3.3. Actin Cytoskeletal Genes Are Upregulated in Aggressive Tumours with Distant Metastasis

Since SPTAN1 is an actin-binding protein with known involvement in cytoskeletal organisation and cell adhesion, we then asked whether expression of cytoskeletal genes and cell adhesion molecules is associated with patient outcomes more generally. To address this question, we computed single-sample gene set enrichment scores for groups of genes involved in actin cytoskeletal function (GO actin cytoskeleton; 503-gene set) [13] and focal adhesion (KEGG focal adhesion; 199-gene set) [14]. Enrichment scores were generated for each tumour sample using the GenePattern algorithm for single-sample gene set enrichment analysis (ssGSEA) (Table S3) [15]. The scores represent the degree to which the individual genes (in the gene sets of interest) are coordinately up- or downregulated within a given tumour sample.

**Figure 2:**
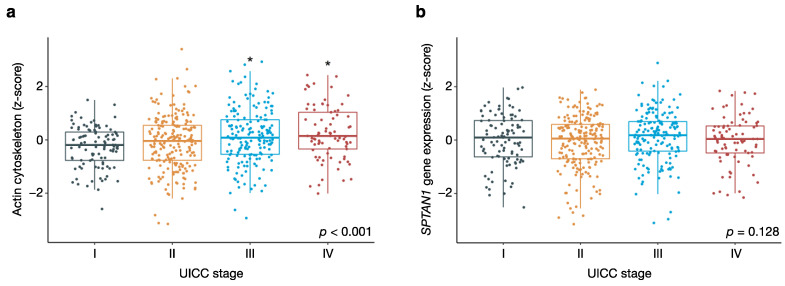
Actin cytoskeletal genes are upregulated in aggressive tumours with distant metastasis. Box plots showing the respective associations between (**a**) UICC stage and actin cytoskeletal gene expression, and between (**b**) UICC stage and *SPTAN1* gene expression. The analysis is based on gene expression data from the TCGA cohort (573 patients). Actin cytoskeletal gene expression in (**a**) was quantified using the GenePattern ssGSEA module. Population means in both (**a**) and (**b**) were compared by one-way ANOVA, and the corresponding *p*-values are shown. Differences between individual UICC stages and the reference group (UICC stage I) were assessed by Dunnett’s post-hoc test, and denoted with an asterisk (*) if they reached statistical significance. Results with a *p*-value < 0.05 were considered statistically significant.

Next, we included the obtained enrichment scores in our proportional hazards model, along with patient age, tumour stage, and SPTAN1 mRNA levels (Table 2). We found that both actin cytoskeletal genes (GO actin cytoskeleton enrichment score) as well as genes involved in focal adhesion (KEGG focal adhesion enrichment score) were independently associated with overall survival, after adjusting for age and tumour stage (*p* < 0.001 for actin cytoskeletal genes; *p* = 0.009 for genes involved in focal adhesion). Like high intratumoural SPTAN1 mRNA levels, coordinate upregulation of genes involved in focal adhesion was associated with better overall survival (Table 2). In contrast to *SPTAN1* and genes involved in focal adhesion, we found that coherent upregulation of actin cytoskeletal genes was associated with poor survival outcomes (Table 2). This may be linked to the progressive increase in actin cytoskeletal gene expression with increasing tumour stage (Figure 2a). Collectively, actin cytoskeletal genes therefore appear to be upregulated in more aggressive tumours with distant metastasis. No association was observed between gene expression and tumour stage for *SPTAN1* alone (Figure 2b).

Overall, we find that upregulation of actin cytoskeletal genes in bulk expression data is associated with increasing tumour stage and poor survival outcomes, whereas increased expression of *SPTAN1* and other genes involved in focal adhesion predicts better overall survival.

### 3.4. Tumour Purity Affects the Expression of Actin Cytoskeletal Genes But Not SPTAN1 Gene Expression

We next sought to clarify the cellular origin of the *SPTAN1* and actin cytoskeletal gene expression signals in our bulk gene expression data. Specifically, we asked whether expression of *SPTAN1* and actin cytoskeletal genes may be related to tumour purity, and whether the transcripts of interest originate from cancer cells or from immune and stromal cells of the tumour microenvironment (TME). To address this question, we computed immune and stromal enrichment scores by ssGSEA algorithm to infer the presence of non-tumour cells in the TME of each tumour sample (Table S3) [16].

**Figure 3:**
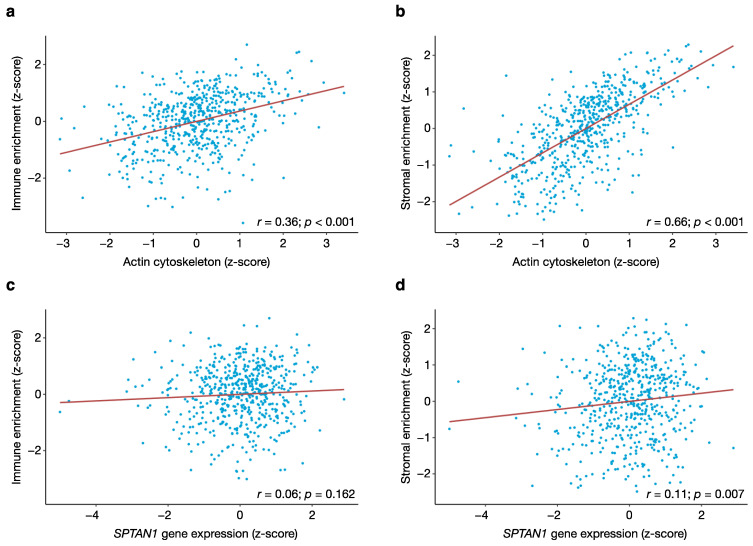
Tumour purity affects the expression of actin cytoskeletal genes but not *SPTAN1* gene expression. Scatter plots showing the respective associations between actin cytoskeletal gene expression and (**a**) immune or (**b**) stromal infiltration, and between *SPTAN1* gene expression and (**c**) immune or (**d**) stromal infiltration. The analysis is based on gene expression data from the TCGA cohort (573 patients). The GenePattern ssGSEA module was used to quantify actin cytoskeletal gene expression, and to compute immune and stromal enrichment scores. Pearson’s *r* coefficient was determined using the cor.test function in the standard R environment, and results with a *p*-value < 0.05 were considered statistically significant.

The expression of actin cytoskeletal genes correlated to varying degrees with both immune and stromal enrichment scores (correlation with immune enrichment score, *r* = 0.36 and *p* < 0.001; correlation with stromal enrichment score, *r* = 0.66 and *p* < 0.001) (Figure 3a,b). From this, we conclude that actin cytoskeletal gene transcripts originate to some extent from both immune and stromal cells of the TME. Conversely, SPTAN1 mRNA levels showed little or no correlation with the immune or stromal enrichment scores (correlation with immune enrichment score, *r* = 0.06 and *p* = 0.162; correlation with stromal enrichment score, *r* = 0.11 and *p* = 0.007) (Figure 3c,d). These latter findings suggest that differential SPTAN1 transcript levels in our bulk expression data reflect tumour cell-intrinsic fluctuations in *SPTAN1* gene expression, rather than variations in the degree of immune or stromal infiltration of the TME.

### 3.5. SPTAN1 Is Related to Cell Adhesion and Polarity, Cytoskeletal Organisation, Motility and Invasion

We next looked for features of tumour biology that might explain why patients with colorectal tumours expressing high levels of SPTAN1 have favourable survival outcomes. To this end, we analysed the 573 TCGA samples by gene set enrichment analysis (GSEA), and applied SPTAN1 mRNA levels (expressed as z-scores) as a continuous phenotype label [18]. We used the GenePattern GSEA algorithm to interrogate the well-curated Hallmark (50 gene sets) [19], KEGG (186 gene sets) [14] and GO (10,271 gene sets) [13] collections from the Molecular Signatures Database [19] (Table S4). For each gene set in the analysis, GSEA provides a normalised enrichment score (NES), nominal *p*-value, and false discovery rate (FDR) to correct for multiple testing.

High levels of *SPTAN1* gene expression were associated with coordinate upregulation of gene sets related to cell polarity (Hallmark apical junction: NES 1.82, *p* < 0.001, FDR = 0.044; Hallmark apical surface: NES 1.66, *p* = 0.010, FDR = 0.098), cell adhesion (KEGG focal adhesion: NES 1.94, *p* = 0.002, FDR = 0.033; KEGG gap junction: NES 1.81, *p* = 0.002, FDR = 0.048; KEGG adherens junction: NES 1.74, *p* = 0.006, FDR = 0.047; KEGG tight junction: NES 1.74, *p* = 0.004, FDR = 0.046), actin cytoskeletal organisation (GO actin cytoskeleton: NES 1.82, *p* < 0.001, FDR = 0.062; GO actin filament organisation: NES 1.85, *p* < 0.001, FDR = 0.057), cell motility and invasion (GO actin filament-based movement: NES 1.75, *p* = 0.010, FDR = 0.078; GO lamellipodium: NES 1.75, *p* = 0.004, FDR = 0.078; GO invadopodium: NES 1.64, *p* = 0.022, FDR = 0.105) (Table 3). These findings implicate SPTAN1 in cellular processes that safeguard cell polarity and epithelial cohesion, and thereby plausibly attenuate tumour aggressiveness. That said, SPTAN1 may also promote a less favourable tumour biology through its involvement in cell motility and invasion.

### 3.6. SPTAN1 Gene Expression Predicts Response to FOLFOX Chemotherapy

Given that the predicted effects of SPTAN1 expression levels on tumour biology did not fully explain why SPTAN1-high colorectal tumours have better survival outcomes, we considered whether the observed survival advantage may result from a favourable response to FOLFOX chemotherapy in the SPTAN1-high group. FOLFOX (folinic acid, fluorouracil, and oxaliplatin) is a standard regimen for treatment of colorectal tumours in both the adjuvant and palliative settings. To address this question, we first applied a qualitative transcriptional signature [20] to the TCGA expression data, allowing us to predict response to FOLFOX chemotherapy for each of the 573 tumour samples (Table S5).

**Figure 4:**
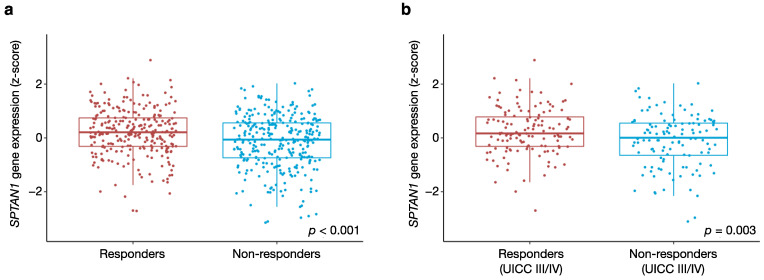
*SPTAN1* gene expression predicts response to FOLFOX chemotherapy. Box plots showing the association between SPTAN1 transcript levels and response to FOLFOX chemotherapy (**a**) in the full TCGA cohort, and (**b**) in the subgroup with advanced (UICC stage III/IV) CRC. A previously established molecular classifier was applied to the TCGA expression data, in order to predict individual patients’ responses to FOLFOX chemotherapy. Mean SPTAN1 transcript levels in predicted responders and non-responders were compared using the *t*-test function in the standard R environment. Results with a *p*-value < 0.05 were considered statistically significant.

Overall, the molecular classifier predicted response to FOLFOX in 265 tumours (46%), versus primary resistance in the remaining 308 tumours (54%). The mean SPTAN1 z-score was +0.20 in responders and −0.18 in non-responders (*p* < 0.001) (Figure 4a). Since systemic therapies are not routinely administered to patients with early-stage tumours (UICC stages I and II), we then focused our analysis on patients with advanced CRC (UICC stages III and IV) who would most likely receive chemotherapy in a real-world setting. Among the 250 patients with stage III and IV CRC, the molecular classifier predicted response to FOLFOX in 132 tumours (53%), versus primary resistance in the remaining 118 tumours (47%). The mean SPTAN1 z-score was +0.25 in responders and −0.11 in non-responders (*p* = 0.003) (Figure 4b). In summary, we demonstrate that high intratumoural SPTAN1 transcript levels are associated with a favourable response to FOLFOX chemotherapy.

**Figure 5:**
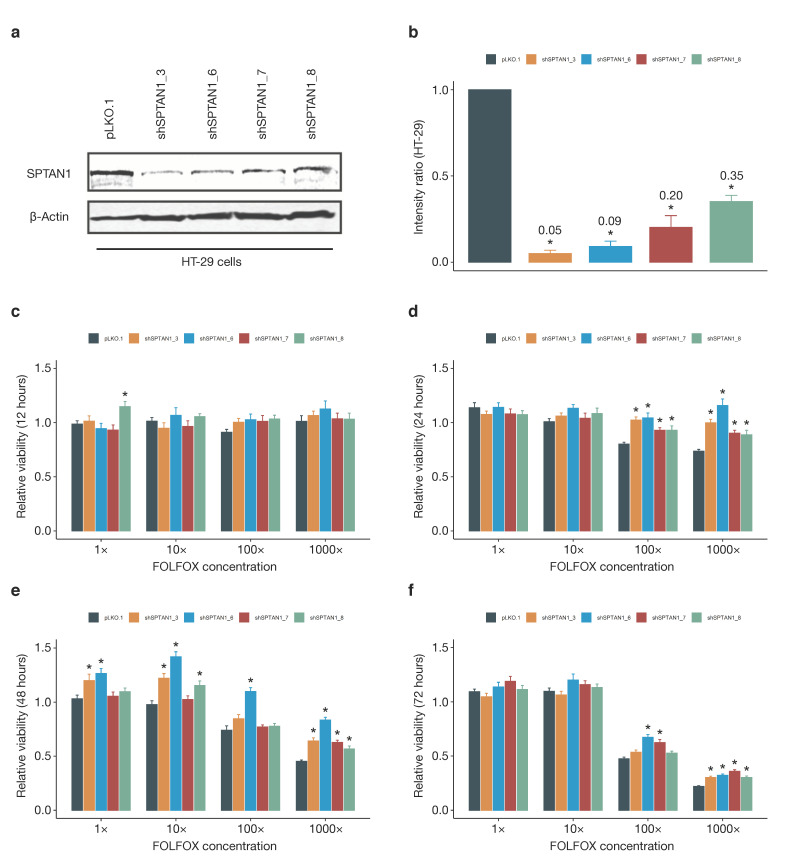
HT-29 cells with SPTAN1 knockdown are less responsive to FOLFOX chemotherapy. Short hairpin RNAs were used to knock down SPTAN1 expression in HT-29 colon cancer cells (**a**). Western blot band intensities were quantified by digital image analysis, to determine SPTAN1 protein levels of knockdown cells relative to control-transduced cells (denoted pLKO.1) (**b**). The uncropped Western blots are shown in Figure S1. Bar charts illustrating the response to FOLFOX chemotherapy in HT-29 colon cancer cells with stable knockdown of SPTAN1 expression (**c**–**f**). Cell viability in response to incremental doses of FOLFOX chemotherapy was measured by tetrazolium dye (MTT) colorimetric assay after (**c**) 12 h, (**d**) 24 h, (**e**) 48 h, and (**f**) 72 h. Data represent averages from three independent experiments, totalling up to 48 individual absorbance readings for each combination of knockdown strain, timepoint and chemotherapy dose. Absorbance readings of FOLFOX-treated cells were normalised to the average absorbance values of the respective control-treated cells. SPTAN1 knockdown cells and control-transduced cells (denoted pLKO.1) were compared by one-way ANOVA. Differences between individual knockdown strains and the reference group (control-transduced cells) were assessed by Dunnett’s post-hoc test, and denoted with an asterisk (*) if they reached statistical significance. Results with a *p*-value < 0.05 were considered statistically significant.

**Figure A1:**
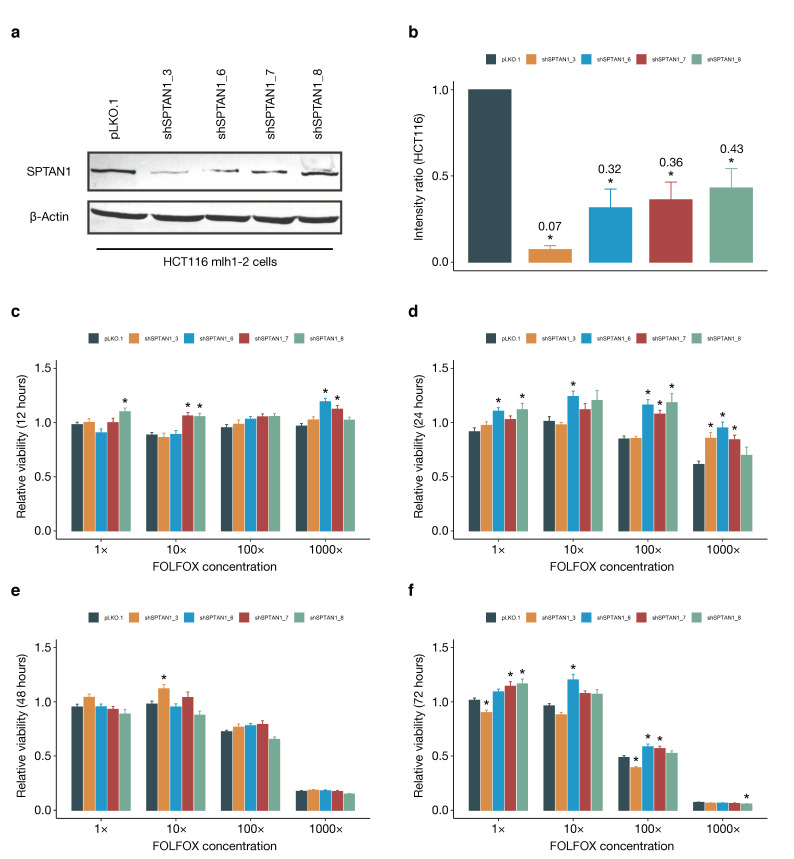
HCT116 mlh1-2 cells with SPTAN1 knockdown are less responsive to FOLFOX chemotherapy. Short hairpin RNAs were used to knock down SPTAN1 expression in HCT116 mlh1-2 colon cancer cells (**a**). Western blot band intensities were quantified by digital image analysis, to determine SPTAN1 protein levels of knockdown cells relative to control-transduced cells (denoted pLKO.1) (**b**). Bar charts illustrating the response to FOLFOX chemotherapy in HCT116 mlh1-2 colon cancer cells with stable knockdown of SPTAN1 expression (**c**–**f**). Cell viability in response to incremental doses of FOLFOX chemotherapy was measured by tetrazolium dye (MTT) colorimetric assay after (**c**) 12 h, (**d**) 24 h, (**e**) 48 h, and (**f**) 72 h. Data represent averages from three independent experiments, totalling up to 48 individual absorbance readings for each combination of knockdown strain, timepoint and chemotherapy dose. Absorbance readings of FOLFOX-treated cells were normalised to the average absorbance values of the respective control-treated cells. SPTAN1 knockdown cells and control-transduced cells (denoted pLKO.1) were compared by one-way ANOVA. Differences between individual knockdown strains and the reference group (control-transduced cells) were assessed by Dunnett’s post-hoc test, and denoted with an asterisk (*) if they reached statistical significance. Results with a *p*-value < 0.05 were considered statistically significant.

**Figure A2:**
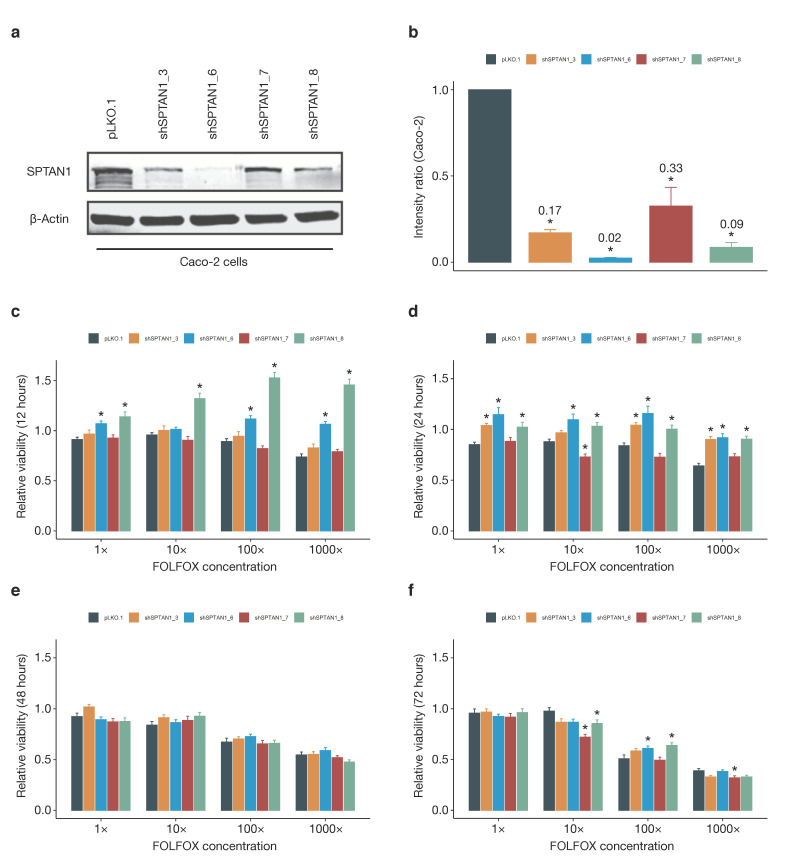
Caco-2 cells with SPTAN1 knockdown are less responsive to FOLFOX chemotherapy. Short hairpin RNAs were used to knock down SPTAN1 expression in Caco-2 colon cancer cells (**a**). Western blot band intensities were quantified by digital image analysis, to determine SPTAN1 protein levels of knockdown cells relative to control-transduced cells (denoted pLKO.1) (**b**). Bar charts illustrating the response to FOLFOX chemotherapy in Caco-2 colon cancer cells with stable knockdown of SPTAN1 expression (**c**–**f**). Cell viability in response to incremental doses of FOLFOX chemotherapy was measured by tetrazolium dye (MTT) colorimetric assay after (**c**) 12 h, (**d**) 24 h, (**e**) 48 h, and (**f**) 72 h. Data represent averages from three independent experiments, totalling up to 48 individual absorbance readings for each combination of knockdown strain, timepoint and chemotherapy dose. Absorbance readings of FOLFOX-treated cells were normalised to the average absorbance values of the respective control-treated cells. SPTAN1 knockdown cells and control-transduced cells (denoted pLKO.1) were compared by one-way ANOVA. Differences between individual knockdown strains and the reference group (control-transduced cells) were assessed by Dunnett’s post-hoc test, and denoted with an asterisk (*) if they reached statistical significance. Results with a *p*-value < 0.05 were considered statistically significant.

To corroborate these findings in a different system, we then determined response to FOLFOX chemotherapy in human HT-29 colon cancer cells with stable knockdown of SPTAN1 expression. Four knockdown strains of the HT-29 cell line were generated using short hairpins targeting SPTAN1 mRNA (shSPTAN1_3, shSPTAN1_6, shSPTAN1_7, shSPTAN1_8), and knockdown efficiency was verified by Western blotting and compared with control-transduced (pLKO.1) cells (Figure 5a,b). Cell viability in response to incremental doses of FOLFOX chemotherapy was measured by tetrazolium dye (MTT) colorimetric assay at four different timepoints (12, 24, 48, and 72 h). In line with our hypothesis, HT-29 cells with reduced SPTAN1 expression were less responsive to FOLFOX chemotherapy compared with SPTAN1-proficient control cells, particularly at higher FOLFOX doses (Figure 5c–f). Similar results were obtained for SPTAN1 knockdown strains of the colon cancer cell lines HCT116 mlh1-2 and Caco-2 (Figure A1 and Figure A2 in Appendix A). On the whole, there was good agreement between SPTAN1 knockdown efficiency and the degree of FOLFOX resistance (Figure 5, Figure A1 and Figure A2).

## 4. Discussion

This study provides evidence of a link between patient outcomes and SPTAN1 expression levels in colorectal tumours. We find that, after taking into account patient age and UICC tumour stage, elevated intratumoural SPTAN1 protein and mRNA levels associate with favourable survival outcomes. Unlike actin cytoskeletal genes, which were more highly expressed in aggressive tumours with distant metastasis and high stromal or immune content, cancer cells themselves appear to account for most of the variation in SPTAN1 expression, and its upregulation is linked to markers of cell-cell contact formation on the one hand, and markers of tumour aggressiveness and invasion on the other hand. Beyond its role in tumour biology, we were able to identify a link between *SPTAN1* gene expression and patient responses to FOLFOX chemotherapy. Overall, this study nominates SPTAN1 as a valuable prognostic marker that could help stratify patients with CRC, and may predict their response to anticancer therapy. Our findings pave the way for future mechanistic studies into the role of SPTAN1 and the actin cytoskeleton in CRC biology and therapeutics.

### 4.1. SPTAN1 as a Prognostic Biomarker

In cancer, reorganisation of the actin cytoskeleton is associated with the epithelial to mesenchymal transition (EMT), tumour aggressiveness and metastatic spread [27,28]. A growing body of evidence supports the view that EMT, cytoskeletal remodelling, and active TGF-β signalling all confer a negative prognosis, both in CRC and across many other types of cancer [12,29,30]. Our data on the upregulation of actin cytoskeletal genes in UICC stage IV tumours are consistent with these general assumptions that place cytoskeletal organisation and remodelling at the heart of tumour biology.

Conversely, the utility of SPTAN1 in determining tumour aggressiveness and predicting patient outcomes is less clear-cut. This is perhaps unsurprising, given that SPTAN1 can assume the role of both tumour promoter and suppressor [11], as reflected in the results of our GSEA analysis. In addition, although alterations in the expression of SPTAN1 have been described in a variety of tumours and tissues [31,32,33,34,35], the directionality of these changes (upregulation vs. downregulation) defies any clear classification [11].

We show here that increased levels of SPTAN1 are associated with longer overall survival times in patients with colorectal tumours. To our knowledge, this is the first study that has identified SPTAN1 expression as a prognostic biomarker in CRC. Our findings are in line with a recent report suggesting that higher *SPTAN1* gene expression levels are associated with better survival outcomes in patients with lung cancer [36], and may prompt similar investigations into the prognostic role of actin-binding proteins in other cancers.

### 4.2. Biological Roles of SPTAN1 in Cancer

We then sought to pinpoint molecular mechanisms that might explain the prognostic benefit of elevated SPTAN1 levels. In our subgroup survival analysis, we saw that the prognostic benefit of high SPTAN1 protein levels was magnified in patients with stage III and IV cancers. This argues that differential expression of SPTAN1 is particularly relevant in advanced CRC and may be directly linked to tumour aggressiveness and metastatic potential in these patients. Importantly, we show that differential SPTAN1 mRNA levels in our bulk expression data are not influenced by stromal content or immune infiltration of the TME, and therefore likely reflect variations in tumour cell-intrinsic processes.

Our transcriptomic pathway analysis indicates that SPTAN1 expression levels may affect tumour aggressiveness and metastatic potential in a context-dependent manner. On the one hand, SPTAN1 appears to be involved in cellular processes which help maintain cell polarity and epithelial cohesion, thus conceivably preventing invasive growth and metastatic spread. This is in line with our previously published in vitro data [8], demonstrating that SPTAN1 knockdown cell lines exhibited weaker cell-cell interactions and may consequently be more likely to detach from the tumour lattice and metastasise. Further supporting this idea, we show here that the coordinate transcriptional upregulation of genes involved in focal adhesion predicts better survival outcomes in patients with CRC. On the other hand, and perhaps in a different cellular context, SPTAN1 may promote tumour cell motility and invasiveness, consistent with the protein’s known role in colon cancer cell migration [7].

Our pathway analysis reinforces the notion that, depending on the cellular context, SPTAN1 can be both a tumour promoter and a tumour suppressor [7,8,11]. The effects of SPTAN1 expression on tumour biology therefore only provide a partial explanation for the favourable survival outcomes of patients with SPTAN1-high CRCs. One possible reason for these seemingly disparate biological roles is that mRNA or protein expression levels are not equivalent to protein function. This is particularly pertinent given that 5% of CRCs harbour *SPTAN1* gene mutations (mostly of unknown significance) [37,38], and because differences in biological function may arise from alternative splicing events of SPTAN1 mRNA and post-translational modifications of the SPTAN1 protein [39,40,41].

### 4.3. SPTAN1 as a Predictor of Therapy Response

Another possible explanation for the prognostic benefit of elevated SPTAN1 levels is that its differential expression affects response to chemotherapy. This appears plausible given that, as stated above, the prognostic benefit of high SPTAN1 protein levels was magnified in patients with stage III and IV cancers—the very patients who would typically receive postoperative chemotherapy. Indeed, we demonstrate that tumours harbouring a gene expression signature predictive of a favourable response to FOLFOX chemotherapy also exhibit higher SPTAN1 mRNA levels. In a complementary in vitro model, SPTAN1 knockdown strains of three widely used colon cancer cell lines were less responsive to FOLFOX chemotherapy compared with SPTAN1-proficient control strains. We therefore suggest that increased SPTAN1 expression sensitises cancers to FOLFOX chemotherapy, thereby contributing to the superior survival outcomes observed in patients with SPTAN1-high tumours.

The molecular mechanisms underlying the association of SPTAN1 levels with chemotherapy response, however, remain to be uncovered. There is some evidence implicating both intact SPTAN1 protein and its caspase-derived cleavage products as pivotal effectors of apoptotic cell death [42,43,44]. It is therefore conceivable that higher pre-treatment levels of SPTAN1 enhance the apoptotic response to FOLFOX chemotherapy, thereby improving survival outcomes. Our findings are consistent with the loss of SPTAN1 expression seen in MLH1-deficient colon cancers that are notoriously unresponsive to standard chemotherapies in the adjuvant setting [45,46].

Importantly, SPTAN1 levels in our study were measured in surgical specimens and prior to the administration of any systemic agents. The timing of SPTAN1 measurements is relevant because chemotherapy demonstrably alters SPTAN1 levels [47,48]. Our observations indicate that SPTAN1 expression levels predict response to postoperative chemotherapy and could be employed as a biomarker to help fine-tune treatment decisions. In a frail elderly patient, for instance, high SPTAN1 levels might justify a more cautious chemotherapeutic regimen, whereas low SPTAN1 levels may warrant a more aggressive treatment approach.

## 5. Conclusions

Taken together, we identify SPTAN1 as a novel prognostic biomarker in CRC and show that SPTAN1 expression levels may predict patient responses to adjuvant chemotherapy. Despite the continuing advances in high-throughput genomics research, there remains an unmet need for precision medicine tools that are both affordable and directly applicable in a real-world setting. Here we illustrate how assessing SPTAN1 expression levels by routine histopathology could directly impact therapeutic decision-making at the bedside. It will be interesting to see whether our observations can be validated in a prospective setting, whether they can be replicated in other tumour types, and whether they are generalisable to actin-binding proteins other than SPTAN1.

## Figures and Tables

**Table 1:** Overall survival in the Frankfurt cohort analysed by multivariable Cox regression.

Variables	Hazard Ratio (95% CI)	Significance Level (*p*-Value)
SPTAN1 Protein (high vs. low)	0.59 (0.35–1.01)	0.054
Age at Diagnosis (Years)	1.03 * (1.01–1.06)	0.001
UICC Stage II (vs. Stage I)	1.14 (0.54–2.41)	0.737
UICC Stage III (vs. Stage I)	1.01 (0.47–2.18)	0.982
UICC Stage IV (vs. Stage I)	3.45 * (1.72–6.92)	5.0 × 10^−4^

Observations: 182 patients; number of events: 76 deaths; * *p* < 0.05.

**Table 2:** Overall survival in the TCGA cohort analysed by multivariable Cox regression.

Variables	Hazard Ratio (95% CI)	Significance Level (*p*-Value)
SPTAN1 mRNA (z-Score)	0.59 * (0.45–0.76)	7.4 × 10^−5^
Age at Surgery (Years)	1.03 * (1.01–1.05)	0.003
UICC Stage II (vs. Stage I)	1.11 (0.41–3.01)	0.830
UICC Stage III (vs. Stage I)	1.37 (0.50–3.71)	0.538
UICC Stage IV (vs. Stage I)	4.34 * (1.58–11.87)	0.004
Cytoskeletal Genes (z-Score)	2.58 * (1.56–4.26)	2.0 × 10^−4^
Adhesion Genes (z-Score)	0.54 * (0.34–0.86)	0.009

Observations: 544 patients; number of events: 71 deaths; * *p* < 0.05.

**Table 3:** Gene sets associated with high *SPTAN1* gene expression in the TCGA cohort.

Gene Set	NES	*p*-Value	FDR
Hallmark Apical Junction	1.82	<0.001	0.044
Hallmark Apical Surface	1.66	0.010	0.098
KEGG Focal Adhesion	1.94	0.002	0.033
KEGG Gap Junction	1.81	0.002	0.048
KEGG Adherens Junction	1.74	0.006	0.047
KEGG Tight Junction	1.74	0.004	0.046
GO Actin Cytoskeleton	1.82	<0.001	0.062
GO Actin Filament Organisation	1.85	<0.001	0.057
GO Actin Filament-Based Movement	1.75	0.010	0.078
GO Lamellipodium	1.75	0.004	0.078
GO Invadopodium	1.64	0.022	0.105

Gene set enrichment analysis (GSEA) of 573 TCGA samples; SPTAN1 transcript levels applied as a continuous phenotype label; NES: normalised enrichment score; FDR: false discovery rate.

## Data Availability

Publicly available gene expression data of 573 TCGA colorectal tumours was downloaded from Sage Bionetworks Synapse (syn4978511) in April 2020, along with matching clinical and survival data. This data was uploaded to Sage Bionetworks in 2015 by Guinney and colleagues [12] and can be found at www.synapse.org (accessed 1 April 2020).

## Supplementary Materials

The following are available online at https://www.mdpi.com/article/10.3390/cancers13143638/s1, Figure S1: Western blots showing all bands and molecular weight markers, Table S1: Patient database of the Frankfurt cohort, Table S2: Patient database of the TCGA cohort, Table S3: Details of single-sample gene set enrichment analysis (ssGSEA), Table S4: Details of gene set enrichment analysis (GSEA), Table S5: Details of treatment response prediction analysis.

## Appendix A

Figure A1: HCT116 mlh1-2 cells with SPTAN1 knockdown are less responsive to FOLFOX chemotherapy; Figure A2: Caco-2 cells with SPTAN1 knockdown are less responsive to FOLFOX chemotherapy.
