# Supplementary material for: SPTAN1 Expression Predicts Treatment and Survival Outcomes in Colorectal Cancer
Source: Cancers (Basel). 2021 Jul 20;13(14):3638. doi: 10.3390/cancers13143638 (PMC8305611; doi:10.3390/cancers13143638)
Supplement: Supplementary file 1 [file cancers-13-03638-s001.zip › cancers-1249907-supplementary.pdf]

cancers

# Supplementary Materials: SPTAN1 Expression Predicts Treatment and Survival Outcomes in Colorectal Cancer

Christopher Schrecker, Sophia Behrens, Rebecca Schönherr, Anne Ackermann, Daniel Pauli, Guido Plotz, Stefan Zeuzem and Angela Brieger

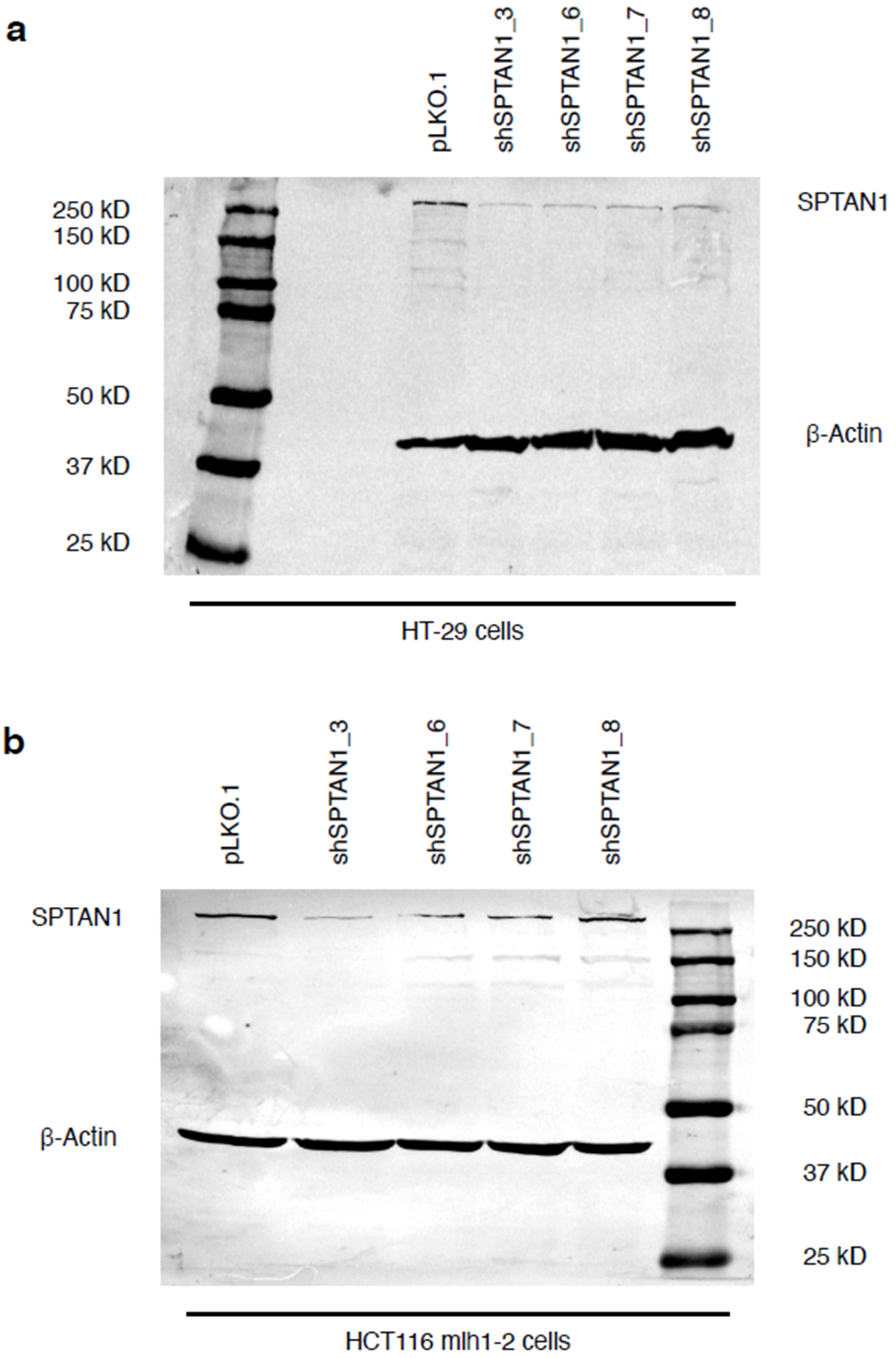

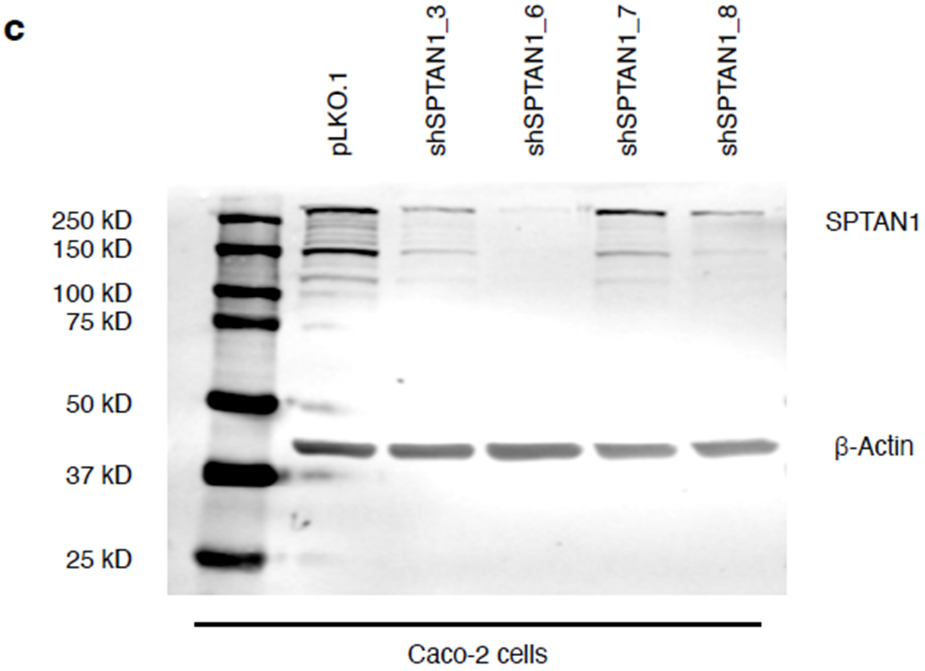


**Figure S1.** The uncropped Western blots.
